# Simulation of Land-Use Spatiotemporal Changes under Ecological Quality Constraints: The Case of the Wuhan Urban Agglomeration Area, China, over 2020–2030

**DOI:** 10.3390/ijerph19106095

**Published:** 2022-05-17

**Authors:** Jingye Li, Jian Gong, Jean-Michel Guldmann, Jianxin Yang, Zhong Zhang

**Affiliations:** 1 Department of Land Resource Management, School of Public Administration, China University of Geosciences, Wuhan 430074, China; jingye.li@cug.edu.cn (J.L.); yangjianxin@cug.edu.cn (J.Y.); zhangzhong@cug.edu.cn (Z.Z.); 2 Department of City and Regional Planning, The Ohio State University, Columbus, OH 43210, USA; guldmann.1@osu.edu

**Keywords:** scenario simulation, land use prediction, ecological protection, Google Earth Engine, Wuhan Urban Agglomeration Area

## Abstract

Human activities coupled with land-use change pose a threat to the regional ecological environment. Therefore, it is essential to determine the future land-use structure and spatial layout for ecological protection and sustainable development. Land use simulations based on traditional scenarios do not fully consider ecological protection, leading to urban sprawl. Timely and dynamic monitoring of ecological status and change is vital to managing and protecting urban ecology and sustainable development. Remote sensing indices, including greenness, humidity, dryness, and heat, are calculated annually. This method compensates for data loss and difficulty in stitching remote sensing ecological indices over large-scale areas and long time-series. Herein, a framework is developed by integrating the four above-mentioned indices for a rapid, large-scale prediction of land use/cover that incorporates the protection of high ecological quality zone (HEQZ) land. The Google Earth Engine (GEE) platform is used to build a comprehensive HEQZ map of the Wuhan Urban Agglomeration Area (WUAA). Two scenarios are considered: Ecological protection (EP) based on HEQZ and natural growth (NG) without spatial ecological constraints. Land use/cover in the WUAA is predicted over 2020–2030, using the patch-generating land use simulation (PLUS) model. The results show that: (1) the HEQZ area covers 21,456 km^2^, accounting for 24% of the WUAA, and is mainly distributed in the Xianning, Huangshi, and Xiantao regions. Construction land has the highest growth rate (5.2%) under the NG scenario. The cropland area decreases by 3.2%, followed by woodlands (0.62%). (2) By delineating the HEQZ, woodlands, rivers, lakes, and wetlands are well protected; construction land displays a downward trend based on the EP scenario with the HEQZ, and the simulated construction land in 2030 is located outside the HEQZ. (3) Image processing based on GEE cloud computing can ameliorate the difficulties of remote sensing data (i.e., missing data, cloudiness, chromatic aberration, and time inconsistency). The results of this study can provide essential scientific guidance for territorial spatial planning under the premise of ecological security.

## 1. Introduction

Land-use/cover change (LUCC) is considered one of the main determinants of global environmental change, and has a significant impact on ecosystems, global biogeochemical cycles, biodiversity, and climate change, due to agricultural and urban expansion and engineering projects [1,2,3,4,5]. Land-use change is an important factor in transforming ecological environment quality via the impact of human activities. Rapid urban expansion and continued economic and population growth have forced dramatic changes in land uses. Continuous changes in land-use patterns have caused regional and even global ecological impacts, such as the urban heat island effect, air pollution, and the loss of arable land, forest area, and biodiversity. Therefore, the study of land-use change is essential for ecological environment protection and sustainable development. The “Land Use and Land Cover Change” program [6] and the “Implementation Strategy of the Land-Use and Land-Cover Change” [7] jointly proposed that the International Geosphere-Biosphere Program provide directions for the study of land-use change. Steffen [8] proposes the concept of “planet boundary” and considers the security threshold of global land system change as its core content. This evaluation shows that the impact of global land-use change on biodiversity has exceeded the planet boundary threshold. The United Nations Sustainable Development Goals (SDG2030) emphasize that land use/cover change plays an important role in the SDG. Creutzig [9] calls for the orderly development, utilization, and management of land to become a global consensus.

The ecological environment refers to the combination of various environmental factors affecting human production, life, and ecosystem development, and is closely related to the sustainable development of society [10,11]. China has among the fastest urbanization rates in the world, and its urbanization rate increased by 42.7% from 1978 to 2019 [12]. However, traditional urbanization has focused too much on development speed, resulting in ecological destruction. As China’s economy and urbanization shift from high-speed growth to high-quality development, the country attaches increasing importance to ecological environmental assessment and protection. The regional ecological quality (EQ) index has been widely used in ecological quality evaluations [13,14,15]. In practical applications, although scholars can adjust the indices and weights differently according to the region under study, they are generally faced with complex evaluation indices, low spatial data accuracy, and infrequent data updates. It is critical to the management of the ecological environment and the sustainable development of society to monitor the status of the ecological environment dynamically, so as to clarify the characteristics and trends of ecological environmental changes [10,16,17,18].

Current land-use and ecological protection models do not fully consider regional ecological security when simulating future land-use patterns. Most of the previous studies have directly selected nature reserves and rivers as the spatial restriction areas of ecological protection, thereby ignoring the role of ecosystem services and landscape integrity in maintaining ecological security. The latter can be defined as the state of the ecological environment that ensures the safety of human life and production as well as the ability to adapt to environmental change. This research assumes that high ecological quality zones (HEQZ) are an effective way to ensure ecological security. Biological movements and ecosystem service flow and transport play a key role in overall ecosystem health and are critical to regional ecological security.

Satellite remote sensing (RS) has the notable convenience of large-area, rapid, and periodically updated observations. It has been widely applied in ecological research, which has improved the assessment of ecological environment quality. To quantify ecological conditions, RS indices have been developed, such as the normalized difference vegetation index (NDVI), the enhanced vegetation index (EVI), the permanent vegetation fraction (PVF), and the drought condition index (DCI) [13,19,20,21,22]. Still, most of these indices are oriented towards a specific ecological-related theme, preventing a comprehensive evaluation of the ecological status of the region. The remote sensing ecological index (RSEI), based on RS information and combining greenness, humidity, dryness, and heat, can help address the above challenges [13,23,24,25,26]. RSEI is easy to obtain and calculate without artificial weighting or thresholds [27]. It is an objective, rapid, and simple monitoring and evaluation technology for urban ecological quality. This index is used at different scales to evaluate the spatial-temporal differentiation of ecological quality. However, there are still some problems in applying RSEI. First, RS images generally entail the problem of cloud occlusion, and cloud removal is difficult. Direct cloud removal causes data loss in the cloud occlusion area. Second, the acquisition times of different scenes and images may be different, making it difficult to splice and lacking in comparability. Small-scale studies usually select data for a few time points in a small area with less cloud cover, while large-scale and long-term time-series studies in areas with more cloud cover are relatively rare. The Google Earth Engine (GEE) platform provides a powerful processing platform for RS data [28,29,30]. GEE-based image processing can ameliorate the problems of RS missing data, cloudiness, chromatic aberrations, and time inconsistencies. In RS research with a large spatial range and long time series, GEE has more advantages, significantly shortening the image processing time and improving work efficiency [31,32,33].

Many land-use change models do not fully consider ecological security constraints in spatial simulation. Most previous studies directly select high-quality cultivated land or other important ecological areas as the spatial restrictions for ecological protection, but this determination of ecological constraint maps is subjective and unscientific. Compared with existing protected areas, rapid and accurate identification based on RS is an effective way to protect ecologically important land and improve the quality of urban residents’ living environment [34]. In recent years, the identification of HEQZ has undergone rapid development, mainly through three methods [35]: (1) natural selection of woodland or nature reserves; (2) landscape patches with adequate ecosystem services and ecological value; (3) establishment of an index system for comprehensively evaluating the importance of ecological patches. However, only a few studies have identified high-quality ecoregions based on RS at large scales.

The Wuhan Urban Agglomeration Area (WUAA) development can be regarded as the epitome of urbanization in China. Establishing strategic areas for ecological protection and identifying HEQZs, using image data processing and RSEI calculation based on the GEE platform, will help construction and green development in the area and provide a benchmark for other similar areas.

This study (1) uses the GEE platform to extract annual RS ecological indices from Landsat RS images of the WUAA obtained over 2000–2020 to delineate high ecological quality zones in the WUAA; (2) monitors the spatial and temporal land-use changes in the WUAA from 2000 to 2020; and (3) simulates land-use changes from 2020 to 2030 under ecological quality constraints, in order to simulate land-use change in the HEQZ areas.

## 2. Materials and Methods

### 2.1. Study Area and Data Preprocessing

**Figure 1:**
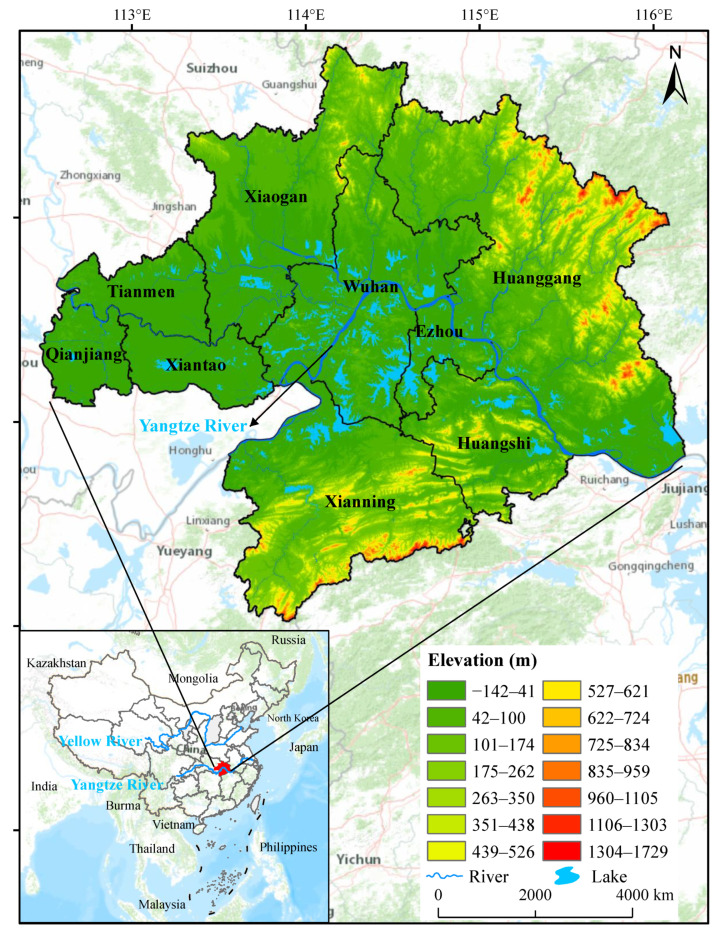
Location of Wuhan Urban Agglomeration Area (WUAA).

WUAA is located at 112°30′ E–116°07′ E, 29°05′ N–31°51′ N in central China, the middle reaches of the Yangtze River, and the eastern part of Hubei Province. WUAA covers nine cities, including Ezhou, Huanggang, Xiaogan, Xianning, Xiantao, Qianjiang, and Tianmen (Figure 1). It is the largest urban area in the middle reaches of the Yangtze River and the most industrial-intensive area in Hubei Province. By the end of 2019, the total area of the WUAA reached 57,943.917 km^2^, accounting for 31.34% of the province’s total area. The permanent population was 31.925 million, accounting for 53.86% of the province’s permanent population. The total GDP was CNY 2768 billion, or 60.4% of Hubei’s GDP. WUAA has undergone significant changes, notably a significant decrease in cultivated land area and a large expansion of construction land. Land development intensity reached 7.24%, far exceeding the 4.62% benchmark set for 2030. However, land-use efficiency is low, rapid economic development takes place at the cost of ecosystem destruction, and the degree of sustainable use needs to be strengthened.

The data required for this study are as follows: (1) Land-use data of the WUAA are derived from Landsat-8 in 2000, 2010, and 2020. According to the LUCC classification system, land can be divided into nine categories: cropland, woodland, grassland, rivers, lakes, artificial wetland, marsh wetlands, construction land, and unused land. The complete detection accuracy is 88.82%. (2) The patch-generating land use simulation (PLUS) model requires data on natural and socio-economic driving factors in the WUAA. Among the natural factors, elevation, slope, and slope direction are derived from a geospatial data cloud platform (https://www.gscloud.cn/ (accessed on 1 April 2022)). The World Soil Database provides soil types and organic matter content data (http://westdc.westgis.ac.cn/ (accessed on 1 April 2022)). Annual mean temperature and rainfall data are obtained from the China Meteorological Data Network (http://data.cmd.cn/ (accessed on 1 April 2022)). Among social and economic factors, population and GDP are derived from the statistical yearbook of each city. Distances to rivers, national roads, provincial roads, high-speed roads, railways, county roads, and township roads are obtained from the National Basic Geographic Information Center (https://www.ngcc.cn/ (accessed on 1 April 2022)).

Pretreatment included cloud removal and a water mask. The quality assessment band generated by the C Function of Mask (CFMask) algorithm is used for cloud removal of Landsat images, which increases the integrity of the research by indicating which pixel might be affected by instruments or clouds (http://www.usgs.gov/land-resources/nli/Landsat (accessed on 1 April 2022)). The specific process is as follows: select a pixel covered by cloud shadow, with cloud and medium cloud confidence, and set its pixel value to 0.

### 2.2. Methodology

#### 2.2.1. Modeling Framework

**Figure 2:**
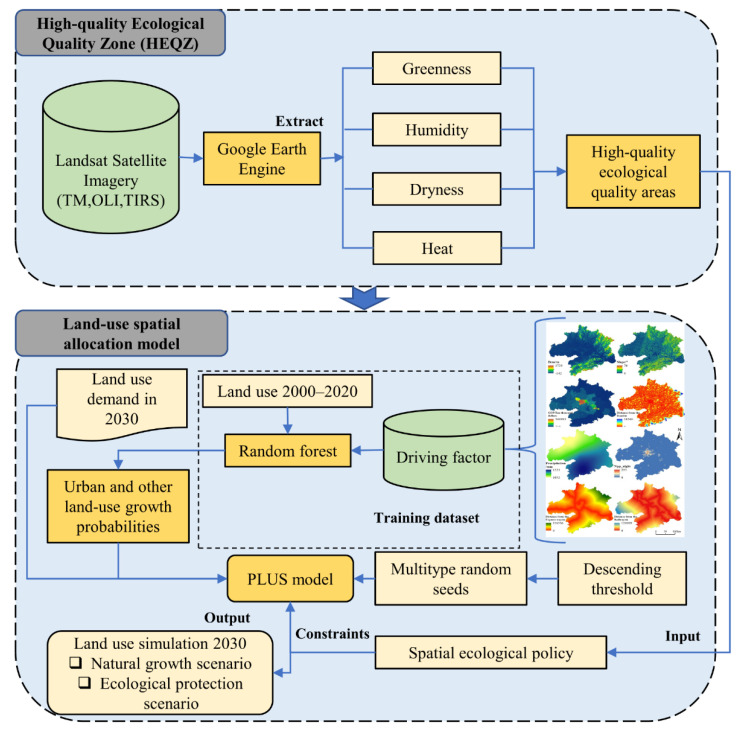
Identification of high-quality ecological areas in the WUAA and framework of land-use simulation (Abbreviations: TM, Thematic Mapper; OLI, Operational Land Imager; TIRS, Thermal Infrared Sensor; PLUS, Patch-generating Land Use Simulation).

The overall framework for identification and simulation modeling, as shown in Figure 2, includes three main steps. First, the RSEI is obtained with the GEE, consisting of the normalized difference vegetation index (NDVI), the moisture index (WET), the surface temperature index (LST), and the dryness index (NDBSI). Second, a reasonable threshold for HEQZ areas is determined by analyzing the RSEI, and the patches with stable and high-quality ecological quality over the past 20 years are extracted and designated as the HEQZ areas in the WUAA. Finally, the HEQZ areas are incorporated into the land-use change simulation model, leading to a comparison of the natural growth and ecological protection scenarios.

#### 2.2.2. Identification of High Ecological Quality Zone Areas

HEQZ is the main tool for maintaining regional ecological security and the overall stability and continuity of the regional ecosystem. HEQZ areas should not only have fundamentally important ecological qualities but also maintain landscape connectivity. Regarding high habitat quality, the identified preliminary patches can be buffered to a certain distance to ensure a direct continuity of ecological patches and promote information exchange and communication between organisms. Therefore, a rapid comprehensive evaluation method is constructed based on the RSEI to identify the HEQZ. To protect the continuity of the landscape, stable patches are extracted, with RSEI greater than 0.7 for all 20 years. The buffer tool in ArcGIS 10.8 is used to buffer these patches 100 m outwards to maintain the continuity of the landscape. The following are the specific methods to calculate the four remote sensing ecological indices based on previous studies [24,36,37,38].

- Greenness index

Urban greenness refers to the area covered by vegetation within the urban area. Vegetation has several ecological service benefits and positively impacts ecological quality. NDVI reflects the plant biomass, leaf area index, and vegetation coverage according to the absorption characteristics of the leaf surface of vegetation in the red band and its reflection in the near-infrared band. Therefore, the NDVI is used to represent the urban greenness index. The calculation method is shown in Equation (1):

(1) $$I_{ndvi}=\left(\rho \mathrm{NIR}-\rho \mathrm{red}\right)\;/\;\left(\rho \mathrm{NIR}+\rho \mathrm{red}\right)$$

where ρNIR and ρred represent the reflectance of the near-infrared and red bands, respectively.

- Humidity index

The WET component based on the Top-hat transform can reflect the surface water conditions, especially the soil moisture state, and WET extraction is carried out based on TM and OLI data. The calculation method is shown in Equations (2) and (3). Before extraction, MNDWI is used to mask the water body. The calculation method is shown in Equation (4); WET reflects the natural land surface humidity.

(2) $$I_{wet\mathrm{TM}}=0.0315\rho \mathrm{blue}+0.2021\rho \mathrm{green}+0.3102\rho \mathrm{red}+0.1594\rho \mathrm{NIR}-0.6806\rho \mathrm{SWIR}1-0.6109\rho \mathrm{SWIR}2$$

(3) $$I_{wet\mathrm{ETM}+}=0.2626\rho \mathrm{blue}+0.2141\rho \mathrm{green}+0.0926\rho \mathrm{red}+0.0656\rho \mathrm{NIR}-0.7629\rho \mathrm{SWIR}1-0.5388\rho \mathrm{SWIR}2$$

(4) $$I_{wet\mathrm{OLI}}=0.1511\rho \mathrm{blue}+0.1973\rho \mathrm{green}+0.3283\rho \mathrm{red}+0.3407\rho \mathrm{NIR}-0.7117\rho \mathrm{SWIR}1-0.4559\rho \mathrm{SWIR}2$$

where ρblue, ρgreen, ρred, ρNIR, ρSWIR1, and ρSWIR2 represent reflectance in bands 1, 2, 3, 4, 5, and 7 of Landsat TM/ETM+ images and reflectance in bands 2, 3, 4, 5, 6, and 7 of Landsat OLI data, respectively.

- Dryness index

Buildings are an essential part of the urban artificial ecosystem, and a building’s impervious surface replaces the original natural ecosystem, leading to the “dryness” of the surface. Therefore, the building bare soil index represents the “dryness.” The built-up index (IBI) and bare soil index (SI) are combined to make up the dryness index, referred to as the normalized difference built-up and soil index (NDBSI). The calculation method is as follows:

(5) $$NDBSI=\left(SI+IBI\right)/2$$

(6) $$SI=\left[\left(\rho \mathrm{SWIR}1+\rho \mathrm{red}\right)-\left(\rho \mathrm{blue}+\rho \mathrm{NIR}\right)]/[\left(\rho \mathrm{SWIR}1+\rho \mathrm{red}\right)+\left(\rho \mathrm{blue}+\rho \mathrm{NIR}\right)\right]$$

(7) IBI=2ρSWIR2ρSWIR1+ρNIR−ρNIRρred+ρNIR+ρgreenρSWIR1+ρgreen2ρSWIR2ρSWIR1+ρNIR+ρNIRρred+ρNIR+ρgreenρSWIR1+ρgreen

where ρblue, ρgreen, ρred, ρNIR, ρSWIR1, and ρSWIR2 represent reflectance in bands 1, 2, 3, 4, 5, and 7 of Landsat TM/ETM+ images and reflectance in bands 2, 3, 4, 5, 6, and 7 of Landsat OLI images, respectively.

- Heat index

Land surface temperature (LST) is an essential component of the Earth’s energy budget and an important parameter reflecting the surface environment. In this study, the inversion surface temperature represents the heat index. Because the Thermal Infrared Sensor (TIRS) sensor of Landsat 8 has two thermal infrared bands, and because TIRS Band 10 is located in a lower atmospheric absorption region than TIRS Band 11, it has higher atmospheric transmittance accuracy. Therefore, bands 6 of Landsat 5 and 10 of Landsat 8 were selected as LST inversion channels. LST used the statistical Mono-Window Model for inversion. The emissivity of the ground object is calculated using vegetation coverage and NDVI. The calculation method is as follows:

(8) L=G×PV+B

(9) Tb=K2/ln(K1/L+1)

(10) $$LST=Tb/\left\{1+\left[\left(\lambda Tb\right)/\rho \right]\ln\varepsilon \right\}-273.15$$

where the grey pixel value *PV* represents the infrared band excursion, while *G* represents the thermal infrared band excursion and *L* the radiation brightness. Equation (9) is a simplified version of Planck’s formula, where *K*1 and *K*2 are calibration parameters. The parameter values are available from the satellite metadata file. ε is the specific infrared emissivity. λ is the central wavelength of the thermal band, and ρ = *s* 1.438 10^−2^ mK.

(11) $$\left\{\begin{array}{c} \in_{water}=0.995\;\left(NDVI\leq 0\right)\\ \in_{building}=0.9589+0.086\times F_{veg}-0.0671\times F_{veg}^{2}\;(0<NDVI<0.7)\\ \in_{natural}=0.9625+0.0614\times F_{veg}-0.0461\times F_{veg}^{2}\;\left(NDVI\geq 0.7\right) \end{array}\right.$$

Vegetation coverage ($F_{veg}$) is based on Landsat NDVI and uses the dichotomy model of mixed pixels. The vertical projection area of vegetation on the ground is compared with the total statistical area:

(12) $$F_{veg}=\frac{NDVI-NDVI_{soil}}{NDVI_{veg}-NDVI_{soil}}$$

where $NDVI_{soil\;}$ is bare land’s normalized difference vegetation index value, and $NDVI_{veg}$ is the normalized difference vegetation index value of complete vegetation coverage. $NDVI_{soil}$ and $NDVI_{veg}$ were selected for $NDVI_{max}$ and $NDVI_{min}$ with a confidence level of more than 95%.

#### 2.2.3. Remote Sensing Ecological Index (RSEI) Evaluation Model

The RSEI model is used to monitor the ecological quality of WUAA from 2000 to 2020. The core is the construction and synthesis of the index system, which comprises greenness, humidity, heat, and dryness, as represented by NDVI, WET, LST, and NDBSI. After normalization, the indices of these four dimensions are integrated into a one-dimensional index by the principal component analysis method, and RSEI is obtained as:

RSEI = *f* (NDVI, WET, LST, NDBSI) (13)

#### 2.2.4. PLUS Model: Land-Use Spatial Allocation

The PLUS model integrates a rule-mining framework based on the land expansion analysis strategy (LEAS) model and a CA model based on multi-type random seeds (CARS) and incorporates the drivers of land expansion. Scenario-specific total amounts of land uses are allocated to grid cells. The PLUS software can be downloaded from https://github.com/HPSCIL (accessed on 1 April 2022).

The total land-use demands affect the local land-use competition. This is captured through a self-adaptive coefficient, which drives the amounts of land use to reach their target demands. The formula for calculating the final probability of land-use type *n* at location *m* and iteration *t* is as follows:

(14) $$FP_{m,n}^{t}=P_{m,n}\times \Omega_{m,n}^{t}\times A_{n}^{t}$$

where $P_{m,n}$ represents the growth probability of land-use type *n* at location *m*. $A_{n}^{t}$ is a self-adaptive coefficient that depends on the gap between the current amount of land-use *n* at iteration 𝑡 and the target demand (see Equation (18) for more detail). $\Omega_{m,n}^{t}$ characterizes the degree of interaction between the surrounding pixels and land-use *n* (grid central pixel), with:

(15) $$\Omega_{m,n}^{t}=\frac{con\left(b_{m}^{t-1}=n\right)}{N\times N-1}\times w_{n}$$

where $con\left(b_{m}^{t-1}=n\right)$ represents the total number of grid cells occupied by land-use *n* at the last iteration within the *N* × *N* Moore window, and $w_{n}$ is the weight matrix for land-use n (different neighborhood effects for different land uses). Model users can define $w_{n}$. In this study, a 3 × 3 Moore window is used, and the number of iterations is set at 300.

The core of the adaptive inertia competition mechanism is the adaptive inertia coefficient $A_{n}^{t}$. Based on the difference between land-use demand and allocated land use in each iteration, this coefficient is automatically adjusted in the next iteration so that the amount of each land-use type is closer to the target demand, with:

(16) $$A_{n}^{t}=\left\{\begin{array}{c} A_{n}^{\theta -1}\;if\;\left|D_{n}^{\theta -2}\right|\leq \left|D_{n}^{\theta -1}\right|\\ A_{n}^{\theta -1}\times \frac{D_{n}^{\theta -2}}{D_{n}^{\theta -1}}\;if\;0>D_{n}^{\theta -2}>D_{n}^{\theta -1}\\ A_{n}^{\theta -1}\times \frac{D_{n}^{\theta -1}}{D_{n}^{\theta -2}}\;\;if\;D_{n}^{\theta -1}>D_{n}^{\theta -2}>0 \end{array}\right.$$

where $D_{n}^{\theta -2}$ and $D_{n}^{\theta -1}$ are the differences between the amounts of land-use *n* at the θ−1 and θ−2 iterations. A roulette wheel is constructed to represent land-use overall probabilities and is used in the next iteration.

A multi-type random seeding mechanism, based on threshold descent, is used to simulate the patch evolution of multiple land-use types. This mechanism generates change ‘seeds’ on the probability surface $P_{m,n}$ with a Monte Carlo approach when the neighborhood effects of a land-use *n* is equal to 0:

(17) $$FP_{m,n}^{t}=\left\{\begin{array}{c} P_{m,n}\times \left(S\times \varepsilon_{n}\right)\times \Omega_{m,n}^{t}\times A_{n}^{t}\;ifA_{n}^{t}=0\;and\;r<P_{m,n}\\ P_{m,n}\times \Omega_{m,n}^{t}\times A_{n}^{t}\;all\;others \end{array}\right.$$

where S is a random value ranging from 0 to 1; $\varepsilon_{n}$ is the threshold for generating the new land-use patches for land-use *n*, as determined by model users. The seeds may generate a new land-use type and grow into new patches formed by a set of cells with the same land-use type. These patch-based models randomly determine the size of new patches, and each patch is separately processed and independently grown. If a new land-use type wins in a round of competition, a decreasing threshold 𝜏 is employed to assess the candidate land-use type 𝑐 that is selected by the roulette wheel:

(18) $$\mathrm{IF}\;\sum_{j=1}^{n}\;\left|G_{c}^{t-1}\right|-\sum_{j=1}^{n}\;\left|G_{c}^{t-1}\right|\;<\;\mathrm{Step}\;\mathrm{Then},\;d=d+1$$

(19) $$\left\{\begin{array}{c} Change\;P_{i,c}>\forall \;and\;T_{n,c}=1\\ \;\;Unchange\;P_{i,c}\leq \forall \;and\;T_{n,c}=0\; \end{array}\right.\;\forall =\mu^{d}\times r$$

where 𝑆𝑡𝑒𝑝 is the step size of the PLUS model to approximate the land-use demand; μ is the decay factor of decreasing threshold ∀, which ranges from 0 to 1 and is set by an expert; 𝑟 is a normally distributed stochastic value with a mean of 1, ranging from 0 to 2; and 𝑑 is the number of decay steps. $T_{n,c}$ is the transition matrix that defines whether land-use type *n* is allowed to convert to type 𝑐. Using this decreasing threshold, the cells with higher overall probabilities are usually the most likely to change. CA models with threshold descent rules are spatiotemporally dynamic, which allows the new land-use patches to grow and freely develop spontaneously, but under the constraints of the growth probabilities.

**Figure 3:**
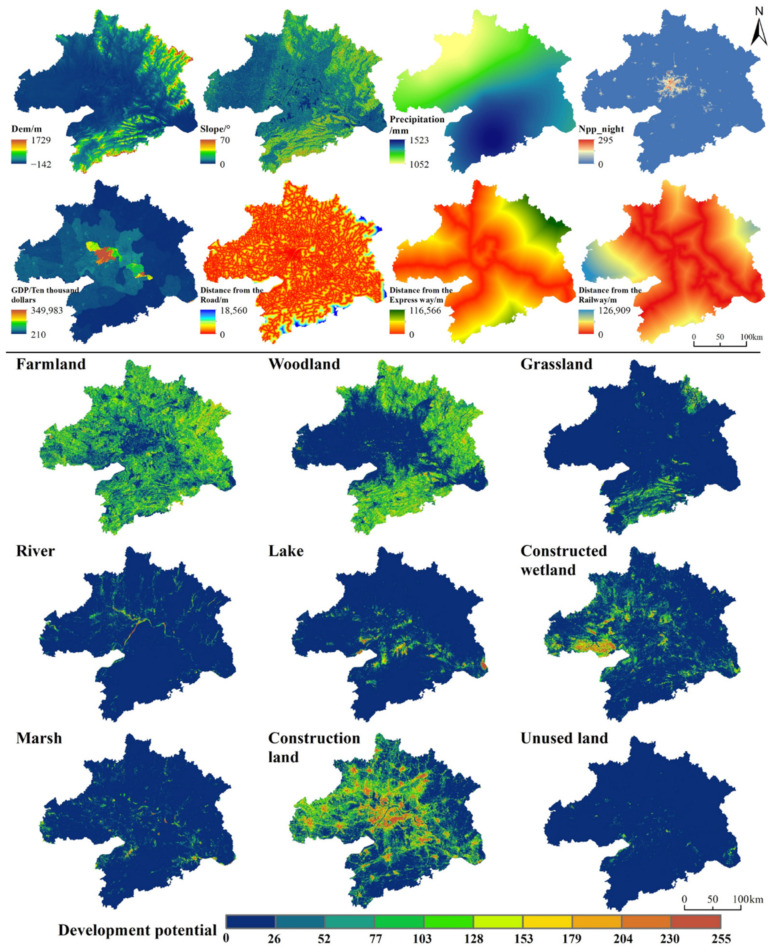
Driving factors for calculating the different land-use probabilities of occurrence in 2020.

Eight spatial variables were selected in the LEAS module to calculate each land-use probability of occurrence from 2000 to 2020 (Figure 3). Natural environmental factors mainly include altitude and slope; climatic factors include precipitation. Socio-economic factors include the night light index and GDP (the night light index can effectively characterize population density). The distance factors include the distances from each point of the study area grid to the city center, county center, railways, and rivers. These distances were calculated using the Euclidean distance tool of ArcGIS in 2000 and 2020. Using the land-use patch changes between 2000 and 2020 and the eight spatial variables, a 15% random sampling was input as the training set of the random forest algorithm. In the CARS module, high ecological quality regions were designated as spatial constraints for ecological protection. Regarding the natural growth scenario, there were no spatial constraints.

#### 2.2.5. Model Validation

**Figure 4:**
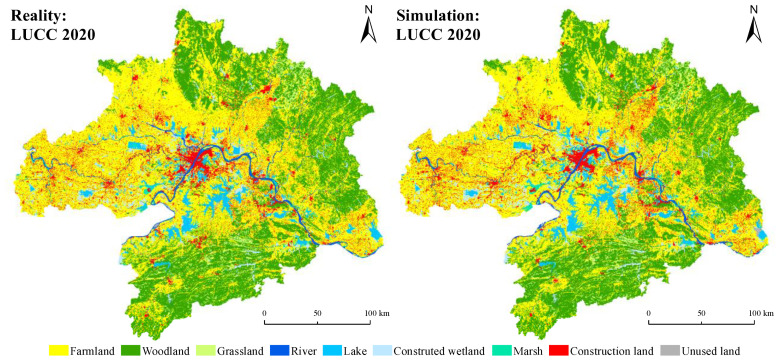
Accuracy of actual and simulated land-use results in 2020 (calculated with Equations (14)–(19)).

The kappa coefficient was used to assess the performance of the PLUS model. Kappa coefficients between 0.6 and 0.8 indicate a high degree of simulation agreement, while 0.8 and 1.0 indicate near-perfect agreement. Taking the land-use data in 2010 as the initial state, the PLUS model predicts land uses in 2020. By comparing actual land-use images for 2020 with the predicted land uses (Figure 4), it was found that the overall accuracy of the simulation was 81.42%, and the individual accuracies for farmland, woodland, and wetlands were all higher than 80%. The PLUS model applied to the WUAA has a high simulation accuracy and therefore is also highly reliable for predicting future land-use changes using validated parameters.

## 3. Results

### 3.1. High Ecological Quality Zone in WUAA

**Figure 5:**
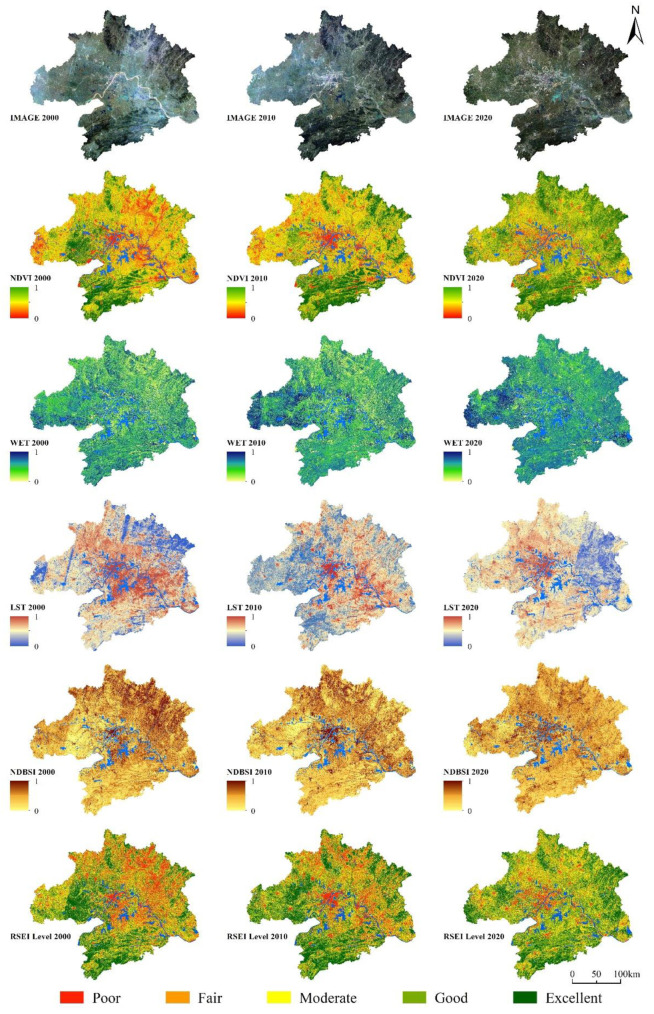
Spatial distribution of ecological quality (calculated with Equations (1)–(13)).

The areas with higher vegetation index values are mainly distributed in the south of WUAA and the northeast hilly area (Figure 5-NDVI). The areas with the highest humidity are mainly located in Tianmen and Qianjiang (Figure 5-WET). The areas with higher surface temperatures are mainly located in the urban area of Wuhan, and the areas with high NDBSI values are mainly located in the northern and central parts of WUAA, accounting for 26.12% of the total area and indicating that the overall regional urban construction intensity is relatively high (Figure 5-NDBSI).

**Figure 6:**
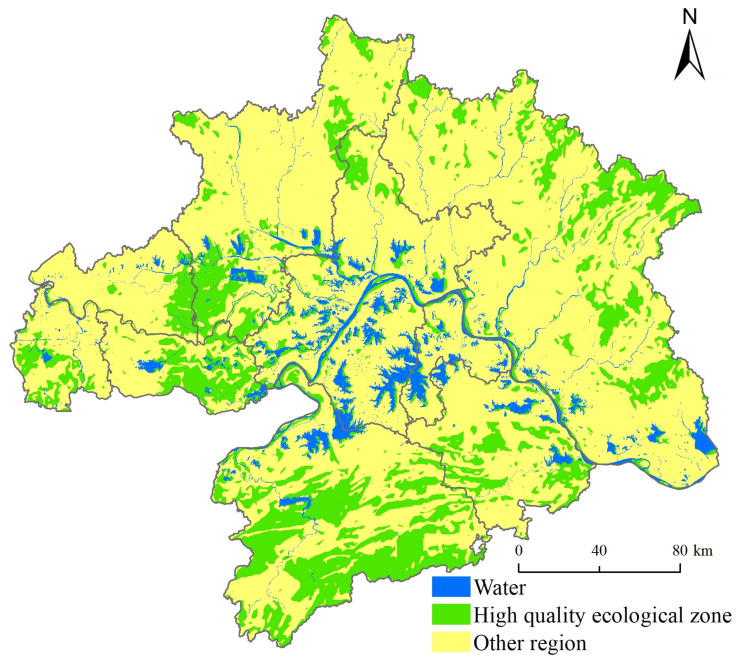
High ecological quality zone in WUAA over 2000 to 2020.

Based on an RSEI value greater than 0.7, the total area of high ecological quality in the WUAA is 21,456 km^2^, accounting for 24% of the total area (Figure 6). The high-quality ecological areas are distributed in the south of Xianning City, the middle of Huangshi City, the east of Huanggang City, and the northeast of Xiaogan City. In terms of land-use types, high-quality ecological areas mainly comprise woodlands, rivers, and constructed wetlands, followed by croplands. As shown in Figure 5, these areas contain many forests, are far away from urban built-up areas, and are located in higher-altitude areas. Some essential ecosystem services, such as water conservation, carbon storage, and biodiversity conservation, are relatively high.

### 3.2. Land-Use Change over 2010–2020

The landscape flow transfer matrices are presented in Table 1 and Table 2. From 2000 to 2020, the total amount of landscape change was 4371 km^2^, with a conversion rate of 7.54%. From 2000 to 2010, this conversion amounted to 4209 km^2^ (7.26%). From 2010 to 2020, the conversion amounted to 5949 km^2^ (10.26%). The largest transfer involved converting farmland into construction land (2.25%), with 1.49% from 2000 to 2010 and 1.82% from 2010 to 2020. The conversion rates from woodland to arable land were 0.77% from 2000 to 2020, 0.32% from 2000 to 2010, and 1.16% from 2010 to 2020. The conversion rate of marsh wetlands was 1.08% from 2000 to 2010 and 0.39% from 2010 to 2020. The outflow of cultivated land and woodland amounted to 4.12% and 1.28%, respectively. In addition, there has been mutual conversion between cultivated land and woodland.

**Figure 7:**
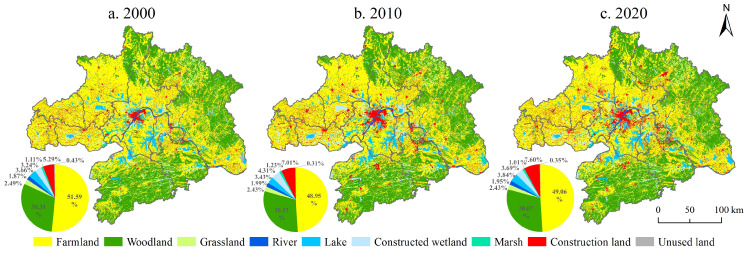
Distribution of land use in WUAA from 2000 to 2020.

The difference between inflow and outflow is used to represent the net flow of the landscape. From 2000 to 2020, rivers, lakes, constructed wetlands, and construction land displayed an inflow trend, with rates of 0.08%, 0.17%, 0.45%, and 2.31%, respectively. Cropland, woodland, grassland, marsh, wetland, and unused land displayed an outflow trend, with rates of 2.53%, 0.24%, 0.06%, 0.10%, and 0.08%, respectively. The net inflow of construction land was the highest from 2000 to 2010 (1.72%) and from 2010 to 2020 (0.59%). The primary inflow sources were arable land and woodland. The newly added construction land is concentrated in Wuhan and Ezhou and scattered in other cities, showing a convergence trend toward the center of the WUAA. The net outflow of cultivated land was the largest, amounting to 2.64% from 2000 to 2010 and 0.11% from 2010 to 2020. The outflow direction mainly includes woodland, constructed wetland, and construction land from 2000 to 2010. The outflow in Wuhan city, Xiantao city, Xiaogan City, and Ezhou city is the largest, followed by Huanggang city. The outflow in Huangshi city is also large. From 2010 to 2020, the inflow came mainly from woodland and construction land, which were distributed in different places (Figure 7).

Based on China’s policy of ecological civilization construction, urban development strategy, permanent essential farmland protection, rigid constraints of construction land control, and other factors, various landscape elements interact and flow in the WUAA. Among them, the scale of cultivated land and construction land has changed dramatically. In the 21st century, the national strategy of urbanization issued a series of policy documents on the coordinated development of significant regions. Such policies promote the development of Wuhan, from the surrounding city to the city center, and thoroughly leverage the advantages of resources agglomeration, radiating and driving efficiency from the city center. The construction of the Optical Valley technology corridor has accelerated the rapid expansion of construction land in Wuhan and Ezhou, occupying a large amount of arable land and woodland. In response to policy requirements, the expansion rate of construction land in the WUAA has slowed down. Since 2002, Hubei province has fully implemented the policy of returning farmland to woodland and grassland, resulting in a net outflow of cultivated land, as manifested by the expansion of woodland and constructed wetland. In 2005, the General Office of the State Council issued the Measures for the Assessment of Cultivated Land Protection Responsibility Targets of Provincial Governments, requiring that cultivated land ownership and protected areas of bare farmland be assessed every five years in each province.

### 3.3. Spatial Changes in Land Uses over 2020–2030

**Figure 8:**
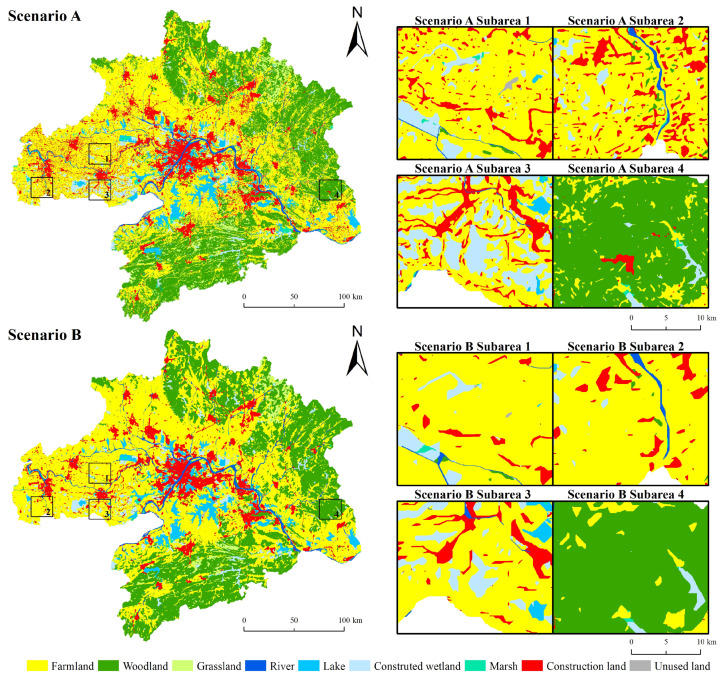
Comparison of Natural Growth (**A**) and Ecological Protection (**B**) scenarios.

Figure 8 displays land-use patterns for the two scenarios (NG: natural growth; EP: ecological protection) in 2030. In both cases, construction land in the WUAA is mainly distributed in Wuhan, Ezhou, and Huanggang. Forests are concentrated in the south and northeast, and cropland is widely distributed in the west and cities surrounding Wuhan. Under the NG scenario, cropland, woodland, rivers, artificial wetlands, marsh wetlands, and construction land in the environmental protection area amount to 6589, 21,553, 235, 3277, 2865, and 5865 km^2^, respectively. Under the EP scenario, the HEQZ protects additional 580 km^2^ of woodland, 624 km^2^ of marsh wetlands, and 235 km^2^ of rivers.

The newly added construction land data over 2020–2030 was extracted under the two scenarios and superimposed over high-quality ecological areas (Figure 8). The newly simulated construction land in the NG scenario is widely spread out throughout the study area’s central cities and eastern parts. Under the EP scenario, the newly added construction land was reduced to 838 km^2^. These results show that ecological protection scenarios based on high-quality ecological regions positively impact environmental protection.

## 4. Discussion

### 4.1. Land-Use Simulation under Ecological Quality Constraints

Previous studies have focused on the impact of land uses on ecosystems, such as how the loss of woodlands and wetlands and the growth of built-up lands can lead to a reduction in ecosystem carbon storage. However, critical high-quality ecoregions have been identified in advance, and local legal planning has been treated as far more important than land management following ecological damage. Increasingly, countries and regions have begun to emphasize the importance of environmental protection planning and urban development land-use decisions to minimize the negative impacts of urban construction on the regional ecology. Rapid population growth and socio-economic development require more construction land. However, China’s expansion of construction land at the expense of ecological land has outpaced urban population growth. Therefore, it is urgent to prevent high-quality ecological lands from being converted into developed lands. The problem is the identification of priority protected areas. In the face of a relatively large area, how to quickly identify ecological reserves is a challenge. Compared with previous studies [35], this research proposes a faster assessment scheme on a large scale, especially at the national, provincial, and watershed scale. A rapid and convenient technical framework has been developed for extracting high-quality ecoregions using RS information derived from the GEE. This study evaluates regional ecological quality from a new perspective, demarcates high-quality ecological regions, considers ecological protection, and meets SDGs development requirements.

Areas of high ecological quality are considered critical areas for protecting ecosystem stability and biodiversity. However, this study mainly considers natural environmental factors identified by remote sensing identification. The maintenance of ecological security should rely on a comprehensive suite of information sources. In previous studies, existing natural protected areas, rivers, and reservoirs were designated as restricted areas in simulations of urban expansion [39,40]. The identified HEQZ in this study can guide land-use simulations under various scenarios. High-quality ecological zones can be used for ecological planning, regional ecological protection zone designation, and urban growth boundary delineation.

### 4.2. Advantages of Future Land-Use Simulation Models

Most previous studies have used logistic regression in land-use simulation to analyze the relationship between natural and socio-economic spatial variables and land-use patterns, for instance, cellular automata (CA) Markov models. These methods cannot address the nonlinear relationships between land-use change and its determinants. Random forest (RF) is a machine learning algorithm often used to approximate nonlinear functions consisting of various independent variables. Based on accuracy tests and comparisons, the RF model has been found to have high accuracy in this study. It has certain advantages compared with the traditional logistic regression and is more suitable for simulating and predicting complex multi-type land-use changes. The PLUS model is a new model based on cellular automata. This model analyzes the spatial characteristics of various land-use expansions and driving factors between two periods and uses an RF algorithm to sample land-use expansions and calculate the development probabilities of various types of land use individually.

### 4.3. Limitations and Future Prospects

The first limitation is related to RS image quality. This study has assessed and mapped four ecological indicators related to ecological quality: dryness, humidity, heat, and greenness. However, other essential services, such as carbon storage and carbon dioxide concentrations, which may also contribute to (or decrease) regional ecological quality, are not considered. In addition, ecological quality should also include factors related to water conservation, water production, runoff and non-point source pollution, microclimate mitigation, and air quality. In conclusion, the high-quality ecological areas extracted in this study cannot fully reflect the whole local ecological quality capacity and ecological value. Ecosystem trade-offs should be identified and assessed. The trade-offs between ecological services and socio-economic services should also be assessed.

The second limitation is the lack of a scientific basis for determining the ecological quality extraction thresholds. In other words, the best areas for high-quality ecoregions should be identified. This study used an ecological remote sensing index greater than 0.7, and further research should carry out a sensitivity analysis of this threshold. To ensure the connectivity of the landscape, initial patches have been buffered by 100 m outwards. However, it is uncertain whether the resulting areas can meet the regional ecological needs for connectivity, and further research is needed on connectivity planning.

Finally, the selected two land-use forecast scenarios, NG and EP, may be too limited for WUAA’s future development planning. It is imperative to consider multi-scenario simulations. The choice of a future urban development scenario should consider the stage of urban development and the urban layout. The optimization of ecological security in the study area should be analyzed under various scenarios to achieve high-quality development with the protection of the ecological environment. Additional scenarios could include cultivated land protection, farmland protection, and energy sustainability. All these scenarios could provide information for the construction of an optimal future land-use pattern in the WUAA.

## 5. Conclusions

According to the 2000–2020 land-use changes, construction land has expanded at the expense of ecological land. Ecological land lost 5.8% of its area, while construction land increased by 3712 km^2^. This development model will continue to disrupt the entire ecosystem. This study has aimed to prevent the disorderly development of cities by delimiting ecological protection areas. Identifying high-quality protected areas is critical for ecological security-based land-use modeling. By using the GEE platform and remote sensing technology, four ecological aspects—dryness, humidity, greenness, and heat—have been considered, reflecting various goals of ecological security. This rapid identification plays an essential role in controlling soil erosion and land desertification in ecologically fragile areas in the WUAA. Parks and guiding the development of the ecological economy have promoted the rational development and protection of forest land and grassland resources, and the areas of grassland and forest land have increased. With the increased awareness of economic and intensive utilization of construction land, the rigid constraints of space planning, and the promotion of secondary development and utilization of abandoned and idle construction land by local governments, the speed of constructed land expansion has been effectively controlled.

More than 24,000 km^2^ of critical ecological reserves are located in the WUAA, mainly in Xianning, Huangshi, and Xiantao. Through the delineation of high-quality ecological zones, woodland, orchards, water areas, and wetlands are protected; the growth of construction land has slowed down, and all new construction land is located outside the HEQZ. To achieve environmental protection goals, stakeholders and government decision-makers should formulate and implement environmental protection measures based on the ecological protection scenario of high-quality ecological zones, which includes timely delineation of high-quality ecological reserves, strict control of the occupation of natural forests and wetlands, protection of water quality and quantity, and strengthening of green infrastructure construction.

## Figures and Tables

**Table 1:** Landscape flow transfer matrix, 2000–2010 (unit: %).

2000 Landscape Types	2010 Landscape Types									
	Cropland	Woodland	Grassland	Rivers	Lakes	Artificial Wetland	Marsh Wetlands	Construction Land	Unused Land	Outflow
Cropland		0.56	0.04	0.06	0.10	1.08	0.24	1.49	0.03	3.61
Woodland	0.32		0.05	0.01	0.01	0.05	0.01	0.25	0.00	0.69
Grassland	0.02	0.09		0.00	0.00	0.01	0.01	0.03	0.00	0.17
Rivers	0.07	0.00	0.00		0.00	0.01	0.08	0.01	0.00	0.17
Lakes	0.09	0.00	0.00	0.03		0.37	0.21	0.07	0.02	0.80
Artificial wetland	0.16	0.02	0.00	0.04	0.17		0.19	0.06	0.03	0.67
Marsh wetlands	0.15	0.00	0.00	0.14	0.21	0.15		0.03	0.02	0.70
Construction land	0.15	0.02	0.00	0.01	0.03	0.02	0.01		0.00	0.23
Unused land	0.02	0.00	0.00	0.00	0.04	0.05	0.09	0.01		0.22
Inflow	0.96	0.71	0.11	0.29	0.56	1.75	0.82	1.95	0.11	7.26

**Table 2:** Landscape flow transfer matrix, 2010–2020 (unit: %).

2010 Landscape Types	2020 Landscape Types									
	Cropland	Woodland	Grassland	Rivers	Lakes	Artificial Wetland	Marsh Wetlands	Construction Land	Unused Land	Outflow
Cropland		0.95	0.05	0.11	0.14	0.39	0.16	1.82	0.03	3.65
Woodland	1.16		0.15	0.01	0.02	0.07	0.01	0.25	0.00	1.67
Grassland	0.06	0.14		0.00	0.00	0.00	0.00	0.03	0.00	0.23
Rivers	0.10	0.01	0.00		0.04	0.03	0.08	0.01	0.00	0.27
Lakes	0.12	0.01	0.00	0.00		0.17	0.08	0.05	0.01	0.45
Artificial wetland	0.75	0.08	0.01	0.02	0.36		0.12	0.10	0.04	1.47
Marsh wetlands	0.21	0.01	0.01	0.07	0.24	0.12		0.03	0.03	0.71
Construction land	1.33	0.21	0.02	0.02	0.04	0.05	0.02		0.01	1.71
Unused land	0.04	0.00	0.00	0.00	0.01	0.02	0.02	0.01		0.09
Inflow	3.76	1.41	0.24	0.23	0.86	0.85	0.49	2.30	0.12	10.26

## Data Availability

The satellite images used in this study are obtained from https://earthengine.google.com (accessed on 11 April 2022).

## Abbreviations

Cellular automata (CA); CA model based on multi-type random seeds (CARS); drought condition index (DCI); dryness index (NDBSI); ecological protection (EP); ecological quality (EQ); enhanced vegetation index (EVI); Google Earth Engine (GEE); high ecological quality zones (HEQZ); land expansion analysis strategy (LEAS); land surface temperature (LST); land-use/cover change (LUCC); gross domestic product (GDP); C Function of Mask (CFMask); moisture index (WET); natural growth (NG); normalized vegetation index (NDVI); patch-generating land use simulation (PLUS); permanent vegetation fraction (PVF); random forest (RF); remote sensing (RS); remote sensing ecological index (RSEI); Sustainable Development Goals (SDG); Wuhan Urban Agglomeration Area (WUAA).
